# A Downstream Processing Cascade for Separation of Caproic and Caprylic Acid from Maize Silage-Based Fermentation Broth

**DOI:** 10.3389/fbioe.2021.725578

**Published:** 2021-08-30

**Authors:** Maria Braune, Bomin Yuan, Heike Sträuber, Stewart Charles McDowall, Roy Nitzsche, Arne Gröngröft

**Affiliations:** ^1^ Biorefineries Department, DBFZ Deutsches Biomasseforschungszentrum gemeinnützige GmbH, Leipzig, Germany; ^2^ Department of Environmental Microbiology, Helmholtz Centre for Environmental Research–UFZ, Leipzig, Germany

**Keywords:** chain elongation, anaerobic fermentation, medium-chain carboxylates, ultrafiltration, liquid-liquid extraction, biorefinery

## Abstract

Production of caproic and caprylic acid through anaerobic fermentation of crops or residual and waste biomass has been regarded as an alternative to the conventional ways, where plant oils and animal fats are mostly used. The downstream processing of the fermentation broth is a particular challenge since the broth has a highly complex composition and low concentrations of the target products. In this study, the proof-of-principle for a separation cascade for caproic (C6) and caprylic acid (C8) produced in a maize silage-based fermentation process was demonstrated. For clarification of the fermentation broth, a filter press and a ceramic ultrafiltration membrane was used to remove coarse solids and to separate suspended particles and macromolecules from the fermentation broth, respectively. With both techniques, the dry matter content was reduced from 6.8 to 2.3% and a particle-free product solution was obtained. Subsequently, the carboxylic acids were extracted with oleyl alcohol by liquid-liquid extraction with an extraction efficiency of 85% for C6 and 97% for C8. Over the whole cascade, 58% of caproic acid and 66% of caprylic acid were recovered from the fermentation broth into the extract. Among all separation steps, solid-liquid separation with the filter press caused the major part of the product loss of 21% of each carboxylic acid. By using separation equipment with a better solid separation efficiency such as decanter centrifuges or belt filter presses this loss could be minimized.

## Introduction

Medium-chain carboxylic acids (MCCA) are carboxylic acids with six to twelve carbon atoms such as caproic (C6) and caprylic (C8) acid. MCCA are conventionally derived from plant oils and animal fats (Bohnet and Ullmann, 2003; Khor et al., 2017). However, the content of C6 and C8 in these resources is usually very low. For example, palm kernel oil contains 0.2% C6 and 3.3% C8 and coconut oil consists of 0.5% C6 and 7.6% C8 (Mancini et al., 2015; Orsavova et al., 2015). The demand for MCCA has been growing due to their wide range of applications in the food and feed as well as chemical industries. Among others, they can be used as such as antimicrobials (Woolford, 1975), feed additives (Khor et al., 2017; Zhou et al., 2019), or to produce fragrances, pharmaceuticals, lubricants, rubbers, or dyes (Angenent et al., 2016; Chen et al., 2017). Given the large market demand due to their various applications (Khor et al., 2017), anaerobic fermentation of dedicated and waste biomass has been intensively studied as an alternative way to produce C6 and C8 (Kenealy et al., 1995; Steinbusch et al., 2011; Cavalcante et al., 2017; Chen et al., 2017; Khor et al., 2017; Scarborough et al., 2018; Han et al., 2019; Lambrecht et al., 2019). From the aspects of sustainable development, the production of these valuable chemicals by applying local resources is more preferred than using ecologically and socially disputed resources. Lambrecht et al. (2019) developed a fermentation process based on maize silage for MCCA production. In this process, naturally occurring microbiomes (microbial communities) were used for the conversion of lignocellulosic substrates in a non-sterile process without cost-intensive substrate pretreatment. However, the resulting fermentation broth (FB) was characterized by low product concentrations and a heterogenic composition with, e.g., substrate particles and microorganisms besides dissolved compounds. This makes the development of an effective and efficient recovery process of fermentation-based organic acids particularly challenging (Hong and Hong, 2005; López-Garzón and Straathof, 2014; Bekatorou et al., 2016; Li et al., 2016).

C6 and C8 are aliphatic, saturated fatty acids with a molecular weight of 116.16 and 144.22 g mol^−1^, respectively. Their boiling point is at 205 and 240°C, respectively (Yalkowsky et al., 2010). They belong to weak acids with pKa values of 4.88 and 4.89 (Kim et al., 2021) and have water solubility values of around 10.1 and 0.8 g L^–1^ at 30°C for C6 and C8, respectively, in their undissociated form (Yalkowsky et al., 2010). At a slightly acidic or neutral pH value of the fermentation, they are mainly dissociated and thus better water-soluble. The water solubility of the acids decreases with the increasing chain length of the alkyl group of the acid.

Various separation techniques for clarification of FBs as well as the recovery, and concentration of organic acids from FBs have been studied (López-Garzón and Straathof, 2014). To achieve an appropriate purification of the acids, an effective removal of the coarse solids from the FB is necessary as the first step. A pre-clarification step can be carried out with filtration or centrifugation (Lukehurst et al., 2010; López-Garzón and Straathof, 2014; Guilayn et al., 2019). As the acids are dissolved in the liquid phase, the filter cake must be as dry as possible for low product losses in the liquid part of the filter cake.

To remove suspended particles, microorganisms, macromolecules, and colloids from the filtrate or supernatant of the pre-clarification, microfiltration and ultrafiltration (UF) can be used (Zacharof and Lovitt, 2014; Longo et al., 2015; Prochaska et al., 2018; Aghapour Aktij et al., 2020). In general, membrane technologies provide excellent fractionation and separation capabilities and go along with low chemical consumption and low energy requirement (He et al., 2012). Since UF is a pressure-driven process, transmembrane pressure is an important factor affecting the flux as well as the retention. Usually, UF membranes are operated at transmembrane pressures between 2 and 8 bar (Benítez et al., 2009).

Liquid-liquid extraction (LLE) (Kertes and King, 1986; Katikaneni and Cheryan, 2002), electrodialysis (Gyo Lee et al., 1998; Ling et al., 2002) or adsorption (Davison et al., 2004; Li et al., 2009) are recommended as primary recovery techniques to remove the products from the aqueous solution and impurities (López-Garzón and Straathof, 2014).

LLE is a comparably well-investigated method, which is accepted as efficient, economical, and environmentally friendly for the separation of MCCAs (Eyal and Canari, 1995; Alkaya et al., 2009; Keshav et al., 2009; Bekatorou et al., 2016). It is particularly suitable for the recovery of low concentrated substances in aqueous media and if thermal separation is not feasible. However, often only artificial FB or model substances have been used for studies (Eyal and Canari, 1995; Hong and Hong, 2005; Keshav et al., 2009; Kannengiesser et al., 2016; Prochaska et al., 2018). If real FB was used, the downstream cascade for C6 and C8 recovery was not the main focus (Katikaneni and Cheryan, 2002; Huh et al., 2006; Udachan and Sahoo, 2014; Bekatorou et al., 2016; Prochaska et al., 2018). Recovery of MCCAs from the solvent can be accomplished by distillation (Saboe et al., 2018) or back-extraction (Ge et al., 2015).

Furthermore, *in-situ* recovery of MCCAs from the fermenters by membrane-based solvent extraction processes was widely studied (Schlosser et al., 2005; Gössi et al., 2020; Carvajal-Arroyo et al., 2021; Xu et al., 2021). However, a limiting factor of these methods is the membrane used, which restricts the extraction interface due to the confined contact area. This can result in lower throughputs compared to conventional countercurrent LLE. The advantage is that the use of membranes in extraction processes prevents emulsification problems and allows large phase ratios. Thus, the costly solvent phase can be kept to a minimum. Nevertheless, the concentration of MCCAs in the solvent is low after a single extraction step, which in turn makes purification of the acids from the solvent difficult and probably costly (Gössi et al., 2020).

Electrodialysis has been studied to accumulate and concentrate carboxylic acids, but a limitation of this process is usually the sensitivity against pollution of the relatively expensive membranes (Li et al., 2009). Compared to LLE, adsorption has the advantages of low-cost adsorbents, quick recovery, easy phase separation, and easy regeneration of adsorbents. However, the relatively low capacity and selectivity of the adsorbents, as well as fouling in the adsorbent bed, limit the applicability of this method (Li et al., 2009). Moreover, electrodialysis and adsorption are not practicable for complex composed FBs, because the separation processes cannot be selectively controlled.

**FIGURE 1:**
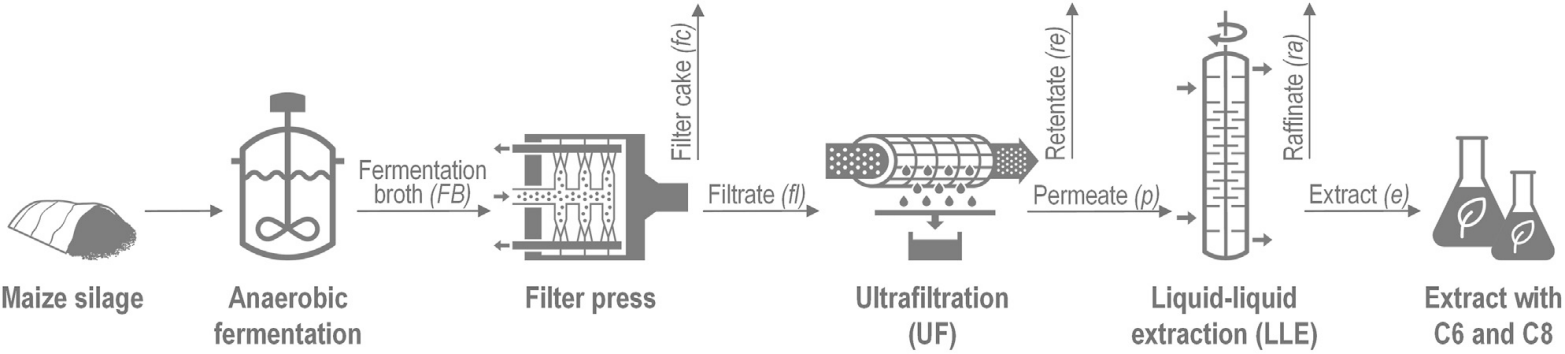
Proposed downstream processing cascade for separation of caproic (C6) and caprylic acid (C8) from fermentation broth obtained by anaerobic fermentation of maize silage.

The aim of the study was to separate C6 and C8 from a maize silage-based fermentation broth with a high overall product yield. The fermentation broth contained biomass residues, various dissolved and undissolved compounds, and low concentrations of the target products. Therefore, a downstream processing cascade including a separation step for removing the solids in order to obtain a particle-free product solution on the one hand and to selectively recover and concentrate the products from the aqueous solution on the other hand was proposed and studied (Figure 1). The reduction of coarse particles in the FB was studied using a filter press, resulting in filter cake and filtrate. To ensure an unobstructed, emulsion-free LLE, UF was studied for the removal of remaining suspended particles from the filtrate. Finding a suitable transmembrane pressure for a high permeate flux and high recovery rates was the aim. A conventional LLE process was then applied to the permeate of the UF with the aim to obtain an extract phase selectively enriched with the target products C6 and C8 at high extraction efficiencies. Oleyl alcohol was chosen as the solvent because it is biocompatible, classified as a non-hazardous substance and has good extraction efficiencies for caproic and caprylic acid compared to other alcohols and esters. The hazard classification is important for the overall concept, since it is possible that residues of the solvent can transfer to the aqueous phase and that these could interfere with relevant biological processes if the residues are further processed, e.g., in biogas or wastewater processes.

## Materials and Methods

### Fermentation Broth

FB originated from two lab-scale MCCA-producing bioreactors that were operated with maize silage as substrate at process conditions similar to those described by Lambrecht et al. (2019). Urea was used as a nitrogen source. The pH value of the broth was 7.1. The broth was collected regularly and stored at 4°C until further use. The concentrations of C6 and C8 were 3.6 and 0.5 g L^−1^, respectively. Because of the high water content (93.3%), the density of the FB was assumed to be 1,000 kg m^−3^.

### Filter Press

A pneumatic basket filter press (GRIFO Macchine Enologiche) was used to separate coarse solids from the untreated FB. The filter press had a working volume of 40 L and consisted of a rubber balloon that was inflated to 3 bar overpressure to force the liquid through a canvas cloth. The filtrate was collected and stored at 4°C until further processing by UF.

To evaluate the separation efficiency in terms of liquids in the solid phase after filter pressing and thus to determine the loss of carboxylic acids, the efficiency of the liquid recovery *E*_*L*_ was calculated as follows:

$$E_{L}=\frac{m_{L,fl}}{m_{L,FB}}\cdot 100\;\%$$ (1)

where *m*_*L,fl*_ is the liquid content in the filtrate and *m*_*L,FB*_ is the liquid content in the untreated FB. Average values based on the results of five runs were determined. The dry matter content calculation is shown in *Analytics*.

### Ultrafiltration

**FIGURE 2:**
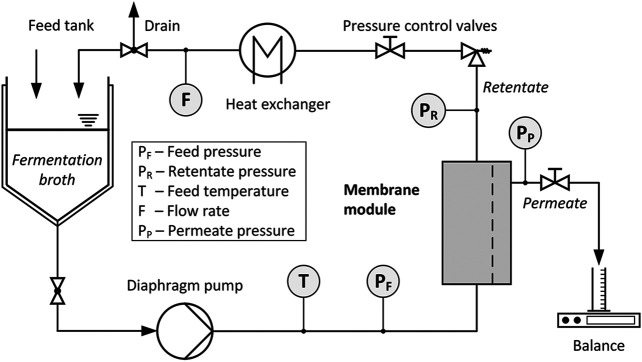
Schematic diagram of the crossflow filtration system.

The filtrate from the filter press was treated by UF. A crossflow filtration system (PS Prozesstechnik GmbH) corresponding to the setup shown in Figure 2 was used. The main components were a 20 L double-jacketed feed tank, a diaphragm pump, a ceramic membrane module (25 mm diameter, 1,178 mm long), and a shell and tube heat exchanger. A commercially available ceramic membrane (atech innovations GmbH) with a molecular weight cut-off (MWCO) of 15 kDa was installed in the membrane module. The materials of the active layer and the support material were ZrO_2_ and α-Al_2_O_3_, respectively. The membrane had seven channels with an inner diameter of 6 mm and a surface area of 0.16 m^2^.

The transmembrane pressure (*Δp*) was adjusted by pressure control valves installed on the retentate and permeate side and measured with pressure sensors. These sensors were installed on the feed (*p*_*f*_), retentate (*p*_*re*_), and permeate (*p*_*p*_) side of the membrane module. The transmembrane pressure was calculated according to Eq. 2:

$$\Delta p=\frac{p_{f}+p_{re}}{2}-p_{p}$$ (2)

The flow rate was regulated with a frequency converter. The temperature was measured with a Pt100 element behind the pump and controlled with a chiller with heating function (Unichiller 025w-H, Peter Huber Kältemaschinenbau AG) connected to the heat exchanger. The permeate flux was determined with an electronic balance (PLS 8000-2A, Kern & Sohn GmbH). During the filtration, measurement data were recorded continuously.

For the determination of an appropriate transmembrane pressure, it was varied between 1 and 4 bar to identify the critical and limiting permeate fluxes. The temperature was set to 40°C since it was close to that of the previous fermentation process. The flow rate was 20 L min^−1^, which corresponded to a crossflow velocity of 1.7 m s^−1^. This was done in total reflux (i.e., both permeate and retentate were recycled to the feed tank) until a steady state was achieved (Nitzsche et al., 2020). At the selected process conditions, in total 82 L of feed solution were concentrated. This means permeate was collected in an external container and retentate was recycled to the feed tank. Since the feed tank of the filtration system had a volume of only 20 L, the volume of the withdrawn permeate was continuously replaced by the same volume of feed solution until the desired volume reduction (*VR*) was reached. *VR* was calculated according to Eq. 3 from the mass of feed (*m*_*f*_) and mass of permeate (*m*_*p*_) as differences in density can be neglected.

$$VR=\frac{m_{p}}{m_{f}}$$ (3)

The permeate flux (*J*_*p*_) through the membrane was obtained at predetermined time intervals and calculated according to Eq. 4.

$$J_{p}=\frac{V_{p}}{t\cdot A_{m}}$$ (4)

where *V*_*p*_ is the volume of the permeate, *A*_*m*_ is the membrane surface area, and *t* is the time. Samples were taken from the feed, retentate, and permeate solution for determination of the observed retention (*R*) as described by Eq. 5.

$$R=\left(1-\frac{c_{p}}{c_{f}}\right)\cdot 100\;\%$$ (5)

where *c*_*f*_ and *c*_*p*_ are the concentrations of the solutes in feed and permeate, respectively.

Between the experiments, the UF membrane was cleaned with a 1 wt% P3-Ultrasil-53 (Ecolab) solution followed by citric acid (≥ 99.5%, IWV Reagents) solution with pH 2.2. Each cleaning step was executed at a transmembrane pressure of 4 bar, a temperature of 40°C, a crossflow velocity of 1.7 m s^−1^, and a duration of 40 min. Backwashing of the UF membrane was not technically possible. After cleaning, the membrane was flushed with deionized water until a conductivity of <10 mS cm^−1^ was reached.

### Liquid-Liquid Extraction

A stirred cell extraction column (PFAUDLER NORMAG SYSTEMS GmbH) was used for LLE. Permeate from the UF process was applied as feed, which was countercurrent to the solvent. Oleyl alcohol was used as solvent in the dispersed phase. The oleyl alcohol (≥ 80%, Merck) had a density of 850 kg m^−3^ and a viscosity of 33 mPas at 20°C. Before extraction, the pH of the FB was adjusted to 5.0 by addition of H_2_SO_4_ (98%, Merck).

**FIGURE 3:**
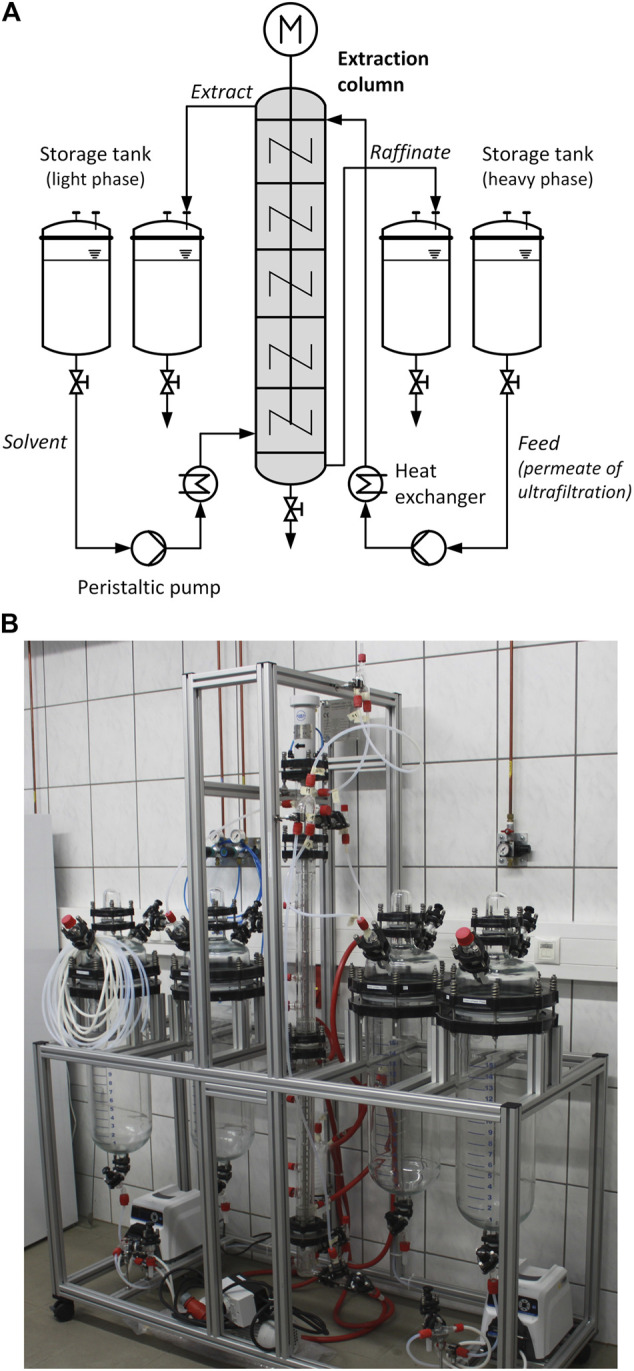
Schematic diagram **(A)** and photo **(B)** of the stirred cell extraction column.

After intensive mixing and mass transfer in the countercurrent stream, the two phases separated in the settling sections at the top and bottom of the column. The aqueous phase raffinate from the bottom and the extract phase enriched with the product from the top were transferred to the respective tanks. The schematic setup of the LLE is depicted in Figure 3. The total length of the column was 1,600 mm consisting of two column sections with 30 stages each, separated by stator plates (fractional free sectional area = 50%). The active mixing zone of the column had a length of 1,200 mm and an internal diameter of 26.4 mm. Each stage was equipped with a six-blade turbine agitator (6 mm rotor diameter, 4 mm rotor height) driven by an electric motor (FMS711-4, Dertec). The internal parts of the column were made of stainless steel.

To start an experiment, the column was filled with the continuous phase and the flow rate was adjusted to the desired value. Then, the agitator and the dispersed phase flow were started. The experiments were carried out at a stirrer speed of 500 rpm. The temperature was kept at 38°C, which corresponded to the temperature of the fermenter. The volume flows of the dispersed and continuous phases were adjusted to 1.3 and 4.5 L h^−1^, respectively. Each experiment ran for at least 30 min until steady state was reached. Afterwards, samples were taken from the raffinate phase in the outlet flow.

To evaluate the extraction process, the transfer of target products from the continuous phase to the dispersed phase was determined by calculating the extraction efficiency (*E*) as follows:

$$E=\left(1-\frac{c_{ra}}{c_{f}}\right)\cdot 100\;\%$$ (6)

where *c*_*f*_ is the initial carboxylic acid concentration in the feed, and *c*_*ra*_ is the concentration in the raffinate after extraction. The carboxylic acid concentration in the extract (*c*_*e*_) was calculated according to Eq. 7.

$$c_{e}=\frac{Q_{f}}{Q_{e}}\cdot \left(c_{f}-c_{ra}\right)$$ (7)

where *Q*_*f*_ and *Q*_*e*_ are the volumetric flow rates of the feed and extract, respectively, and *c*_*f*_ and *c*_*ra*_ are the carboxylic acid concentrations in the feed and the raffinate, respectively.

The column loading (*B*) was calculated as follows:

$$B=\frac{\left(Q_{e}+Q_{f}\right)}{A_{C}}$$ (8)

where *Q*_*e*_ and *Q*_*f*_ are the volumetric flow rates of the extract and feed phase, respectively, and *A*_*C*_ is the cross-section area of the column.

### Analytics

Determination of the dry matter content was carried out in triplicate according to DIN EN 12880:2000-02 (Characterization of sludges, 2001). For this, a sample of 10–60 g was placed in a ceramic crucible and dried at 105°C for 24 h. The dry matter (*DM*) of the sample was calculated as weight percentage according to Eq. 9.

$$DM=\frac{m_{c}-m_{a}}{m_{b}-m_{a}}\cdot 100\;\%$$ (9)

where *m*_*a*_ is the mass of the crucible, *m*_*b*_ is the mass of the crucible with sample before drying and *m*_*c*_ the mass of the crucible with sample after drying.

The turbidity of filtrate and permeate was determined using a spectrophotometer (DR 3900, Hach). The measurement was carried out at a wavelength of 860 nm in a range of 40–600 NTU according to DIN EN ISO 7027 (Water quality, 2000). The samples were measured in triplicate and undiluted.

The carboxylic acid concentration in the aqueous solution was analyzed using a 7890 A gas chromatograph with a DB-FFAP column (60 m × 0.25 mm × 0.5 µm) and a flame ionization detector (FID) from Agilent Technologies. The analytes were esterified by adding 0.5 ml of methanol and 2.5 ml of sulfuric acid (20%) to 0.3 ml of the sample along with 1.7 ml of deionized water and 1 ml of 2-methylbutanoic acid (184 mg L^−1^) as an internal standard. The samples were analyzed in triplicate. Detailed information about parameters of the gas chromatograph were already published by Schumacher et al. (2019).

## Results and Discussion

### Filter Press

First, the FB was subjected to a solids removal process through a filter press. The dry matter content of the FB was reduced from 6.8 to 2.5% in the filtrate. The filtrate was a turbid liquid, therefore the turbidity value was outside the measurement range. The dry matter of the filter cake was 23% after pressing. The mass fraction of filtrate and filter cake was 79 and 21%, respectively. In the filtrate, a liquid recovery with an efficiency of 83% was reached. The filtrate contained a total of 98% of liquids. During this process, the carboxylic acid concentrations in the liquid phases remained unchanged. The filter cake consisted of 77% of liquids, this resulted in a loss of 21% of the total amount for each acid.

Separation with the filter press was found to be the step with the highest carboxylic acid loss since a considerable amount of liquid containing carboxylic acids were retained in the filter cake. This process step could be improved by process or equipment optimization. For example, flushing the filter cake with process water, e.g., with retentate from the UF step or raffinate from the LLE step may enhance the MCCA recovery. The used filter press applied in this work is only applicable on a laboratory scale. On a larger scale, plate and frame filter or belt filter presses would probably be more suitable, as both provide cake washing (Tarleton and Wakeman, 2007; Perlmutter, 2015). Additionally, it was proven that commercial separators such as belt presses and decanter centrifuges have a dry matter removal of approximately 60%. Studies have shown that the addition of polymeric flocculants prior to centrifugation can improve partitioning and further improve dry matter removal from 51–53% to 65–71% (Lukehurst et al., 2010).

### Ultrafiltration

**FIGURE 4:**
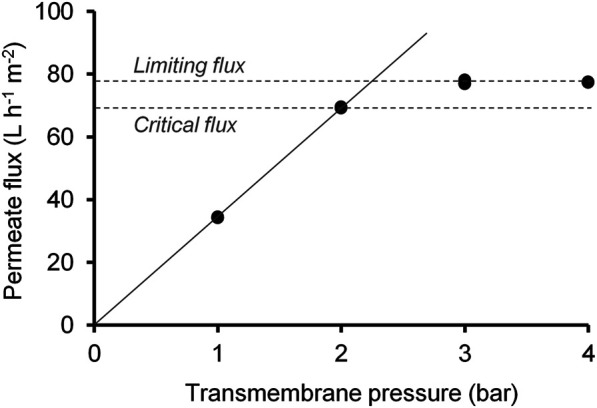
Influence of transmembrane pressure on permeate flux and determination of critical and limiting permeate flux.

For the removal of suspended solids and a part of soluble macromolecules from the filtrate, UF was used. The critical flux, an important factor in making the UF process as energy efficient as possible, was achieved between a transmembrane pressure of 2 and 3 bar (Figure 4). In this range, a permeate flux of 70–80 L m^−2^ h^−1^ could be attained. Beyond this critical flux, no further significant increase of flux was achieved due to compression and deformation of accumulated particles on the membrane surface (Waeger et al., 2010). The deviation from the linear increase of the flux with transmembrane pressure higher than 2 bar thus indicated the transition to the limiting flux as a cause of concentration polarization and gel/cake layer formation. Deposition cannot be avoided, but the choice of a suitable transmembrane pressure value allows mass accumulation to have a small impact on process efficiency. Different studies tested the effect of transmembrane pressure on the permeate flux for filtration of digestate of anaerobic digestions. Thereby, the critical transmembrane pressure can be very low (Elmaleh and Abdelmoumni, 1997; Waeger et al., 2010; Gienau, 2018). Waeger et al. (2010) tested two UF membranes, including a comparable membrane with a MWCO of 20 kDa. They observed no further increase in permeate flux at a transmembrane pressure higher than 0.5 bar, which was between 30 and 40 L m^−2^ h^−1^. In our investigations, a transmembrane pressure of 3 bar was chosen as operating pressure to reach a high permeate flux below the critical level.

**FIGURE 5:**
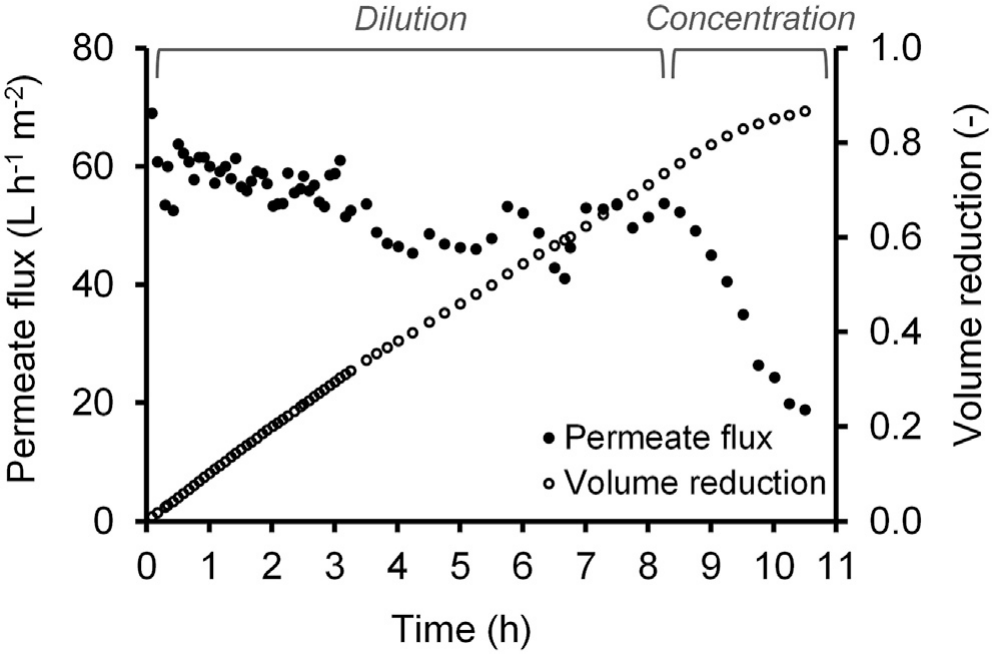
Permeate flux and volume reduction during the concentration of filtrate, dilution due to periodic refilling.

At the determined transmembrane pressure, the filtrate from the filter press was concentrated to a *VR* of 0.87, i.e., 87% of the feed solution was transferred to the UF permeate. The results of permeate flux and *VR* vs. filtration time are depicted in Figure 5. Within the first 1.7 h (*VR* = 0.17), a decrease in the flux of 10% from the initial value of 60 to 54 L m^−2^ h^−1^ was observed. This flux value was maintained until the feed was no longer refilled (*t* = 8.3 h, *VR* = 0.74). The flux decreased over time as the feed became more concentrated due to the increased substances remaining in the retentate. However, a periodic refilling of the feed tank resulted repeatedly in an increase of the flow rates. This fact suggests that the flux decline was related to reversible fouling and an increase in concentration and viscosity. Then, the concentration phase began until filtration was stopped after 10.5 h (*VR* = 0.87) because the feed volume reached the minimum technical level.

The retention of C6 and C8 was negligible, so the concentration of C6 and C8 was unchanged after the UF process. Based on the mass balance, the loss of C6 and C8 in the UF was calculated to be about 13% resulting from the *VR* of 87%.

The turbidity measurement, which was below the lower detection limit of 40 NTU, showed that suspended solids and partly macromolecules were very efficiently separated by the UF. However, this resulted in only a small reduction in dry matter content from 2.5% in the feed solution to 2.3% in the permeate, indicating that most of the impurities consisted of dissolved salts and compounds that did not evaporate at 105°C during the dry matter determination.

A clear, particle-free permeate without carboxylic acid loss was obtained and an average flux rate of around 50 L m^−2^ h^−1^ was achieved. The change of the flux value over time and the long-term stability of the membrane still need to be investigated in future experiments.

### Liquid-Liquid Extraction

**FIGURE 6:**
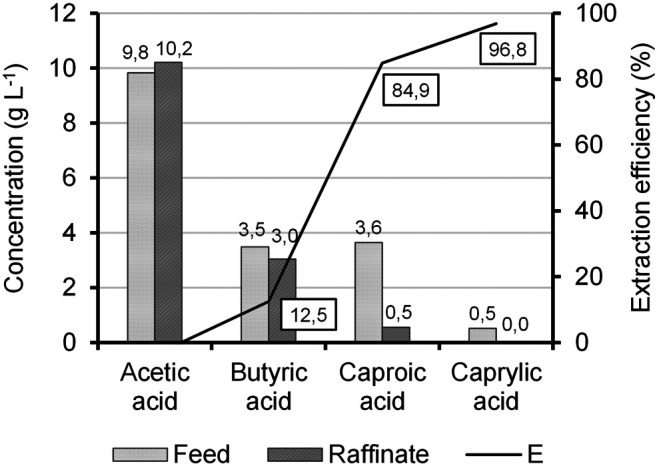
Extraction efficiency (E) and concentrations of different carboxylic acids in the feed and raffinate stream.

LLE was used to separate the carboxylic acids from the aqueous environment in the permeate and to concentrate them selectively in oleyl alcohol as a solvent. Figure 6 shows the concentrations and extraction efficiencies of different carboxylic acids in the feed and raffinate after the extraction process. The extraction efficiency increased with an increase in the chain length of the acids because of a decrease in the polarity of the acids. An extraction efficiency of 85% for C6 and 97% for C8 was achieved. The concentration of the short-chain carboxylic acid acetic acid increased slightly after extraction from 9.8 to 10.2 g L^−1^. The negative extraction efficiency may result from limited measurement accuracy of approximately ± 10% of the GC analysis. Due to its polarity, it can be assumed that acetic acid is not extracted by oleyl alcohol. Butyric acid, in turn, was extracted with an efficiency of about 13%. This indicates high selectivity of the oleyl alcohol for C6 and C8 towards the short-chain carboxylic acids in the FB, which is desired for an effective extraction process.

While the extraction efficiency was high, the achieved C6 and C8 concentrations in the solvent were 10.9 g L^−1^ and 1.7 g L^−1^, respectively (according to Eq. 7). Therefore, the loss of C6 and C8 in the extraction step was calculated as 15 and 3% on the basis of the mass balance, respectively. Thus, the solvent could only be enriched about 3-fold with C6 and C8. One way to increase the yield could be to increase the column loading, which was with 11.18 m^3^ m^−2^ h^−1^ near the flooding point. The flooding point is the hydrodynamic upper absolute load limit of a column, above which correct phase control is no longer guaranteed (Rode et al., 2013). Another possibility could be the variation of the volumetric flow rate ratio ( $\lambda =Q_{d}/Q_{c}$ ) between dispersed and continuous phase, which was 3.5 in this study. An increase in the ratio would increase the acid concentration in the extract, but at the same time the extraction efficiency would decrease and thus the loss of acids in the raffinate would increase. Increasing the energy input through the stirrer speed could also be an option to enhance the yield. As the stirrer speed increases, the droplet size decreases, thus increasing the overall droplet surface area and with it the mass transfer area. However, if the stirrer speed exceeds a critical speed, droplet disintegration is promoted. The resulting lower ascent velocities of the droplets in combination with the higher flow turbulence of the continuous phase lead to an increase in the droplet residence time in the column (Tsouris et al., 1994; Korb and Bart, 2017). This increases the proportion of the disperse phase in the active part of the column and the flooding point is reached earlier, further reducing the possible column loading limit.

Future experiments will investigate to what extent the hydrodynamic properties can be optimized in terms of yield and extraction efficiency. However, the range of application of this column with an inner diameter of 26.4 mm is limited, as strong wall effects occurred with this small column diameter and thus the maximum column loading was almost reached. To achieve better results, a cross-section expansion of the column may have to be considered.

The enriched solvent could be further processed by distillation to recover the solvent and purify the acids. The boiling point of oleyl alcohol is 305–370°C at atmospheric pressure, which is much higher than C6 (205°C) and C8 (240°C), so that the carboxylic acids can be distilled off and oleyl alcohol can be obtained as a sump product to be reused, saving chemicals and thus costs.

### Overall Product Yield

**FIGURE 7:**
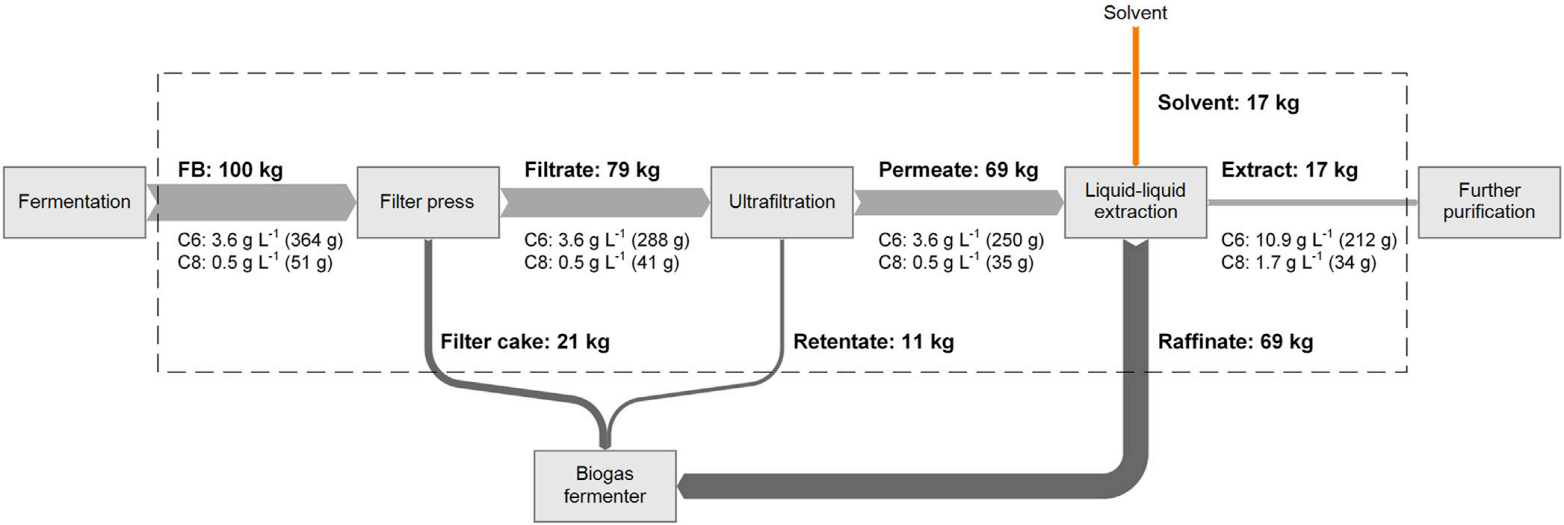
Overall mass balance of the downstream processing cascade including caproic (C6) and caprylic acid (C8) concentrations (FB: fermentation broth).

To evaluate the efficiency of the whole downstream cascade, the overall yield *Y* of C6 and C8 was calculated as follows:

$$Y=\frac{m_{a,out}}{m_{a,in}}\cdot 100\text{\%}$$ (10)

where *m*_*a,out*_ and *m*_*a,in*_ are the mass of the carboxylic acid *a* in the outlet and inlet flow, respectively. To simplify the calculation, the total mass of FB was assumed as 100 kg. The block flow diagram in Figure 7 illustrates the mass balance of every process step in the downstream processing cascade with the corresponding C6 and C8 concentrations. All investigated process steps are illustrated within the dashed box. At the end of the developed downstream processing cascade, a yield of 58% of C6 and 66% of C8 was achieved.

Residual streams of the cascade such as filter cake, retentate, and raffinate still contained fermentable substances and can be further processed into a secondary utilization process, e.g., in a biogas plant to produce biomethane, electricity, or heat.

## Conclusion

A downstream cascade suitable for the purification and recovery of C6 and C8 from maize silage-based FB was developed and tested. The solids were completely removed by using a filter press and an UF membrane with a MWCO of 15 kDa. The dry matter content was reduced from 6.8 to 2.3% and a particle-free product solution was obtained. An average liquid recovery of 83% was achieved with the filter press. However, the dry matter of the filter cake was still only 23% which resulted in a product loss of 21% of C6 and C8 in the remaining liquid in the filter cake. To improve the recovery of this step and validate scalability, trials with alternative equipment such as a belt filter press with backwashing with process water (e.g., raffinate) should be carried out to optimize the process. Due to the maximum achievable volume reduction, the product loss in the UF was 13% for C6 and C8. The average flux value of approx. 50 L h^−1^ m^−2^ could be maintained over the test duration of approx. 10 h. Next, it is necessary to investigate how the flux value behaves in longer test series and what the membrane’s regenerability is. Finally, LLE of C6 and C8 from the water phase reached an extraction efficiency of 85% for C6 and 97% for C8. Oleyl alcohol as a solvent showed a good selectivity for C6 and C8 and most of the polar short-chain carboxylic acids were separated from the MCCA and remained in the raffinate. However, the solvent could be only enriched about 3-fold with C6 and C8. As the column was already operated close to its maximum column capacity, a technical modification to increase the yield may have to be considered. The overall yield of the developed downstream processing cascade was 58% of C6 and 66% of C8.

## Data Availability

The original contributions presented in the study are included in the article/Supplementary Material, further inquiries can be directed to the corresponding author.
