# Corrigendum: A downstream processing cascade for separation of caproic and caprylic acid from maize silage-based fermentation broth

**DOI:** 10.3389/fbioe.2022.1049451

**Published:** 2022-10-12

**Authors:** Maria Braune, Bomin Yuan, Heike Sträuber, Stewart Charles McDowall, Roy Nitzsche, Arne Gröngröft

**Affiliations:** ^1^ Biorefineries Department, DBFZ Deutsches Biomasseforschungszentrum gemeinnützige GmbH, Leipzig, Germany; ^2^ Department of Environmental Microbiology, Helmholtz Centre for Environmental Research–UFZ, Leipzig, Germany

**Keywords:** chain elongation, anaerobic fermantation, medium-chain carboxylates, ultrafiltration, liquid-liquid extraction, biorefinery

In the published article, there was an error. The water solubility and the corresponding temperature of caproic acid quoted in the introduction was incorrect.

A correction has been made to the Introduction, 2nd paragraph. This sentence previously stated:

“They belong to weak acids with pKa values of 4.88 and 4.89 (Kim et al., 2021) and have water solubility values of around 1.1 and 0.8 g L^−1^ at 40°C for C6 and C8, respectively, in their undissociated form (Yalkowsky et al., 2010).”

The corrected sentence appears below:

“They belong to weak acids with pKa values of 4.88 and 4.89 (Kim et al., 2021) and have water solubility values of around 10.1 and 0.8 g L^−1^ at 30°C for C6 and C8, respectively, in their undissociated form (Yalkowsky et al., 2010).”

The authors apologize for this error and state that this does not change the scientific conclusions of the article in any way. The original article has been updated.

